# Pretreatment with ACE inhibitors improves acute outcome of electrical cardioversion in patients with persistent atrial fibrillation

**DOI:** 10.1186/1471-2261-5-3

**Published:** 2005-01-24

**Authors:** Trudeke Van Noord, Harry JGM Crijns, Maarten P van den Berg, Dirk J Van Veldhuisen, Isabelle C Van Gelder

**Affiliations:** 1 Department of Cardiology, Thoraxcenter, University Hospital Groningen, P.O. Box 30.001, 9700RB Groningen, The Netherlands; 2 Department of Cardiology, University Hospital Maastricht, P. Debyelaan 25, 6229 HX, Maastricht, The Netherlands

## Abstract

**Background:**

Persistent atrial fibrillation (AF) is difficult to treat. In the absence of class I or III antiarrhythmic drugs sinus rhythm is maintained in only 30% of patients during the first year after electrical cardioversion (ECV). One of the remodeling processes induced by AF is fibrosis, which relates to inducibility and maintenance of AF. The renin-angiotensin system may play a important role in this. The aim of this study was to investigate the role of angiotensin-converting enzyme (ACE) inhibitor use on efficacy of ECV, and occurrence of subacute recurrences.

**Methods:**

One hundred-seven consecutive patients with persistent AF underwent ECV. In twenty-eight (26%) patients ACE inhibitors had been started before initiation of the present episode of AF ('pre-treated' patients).

**Results:**

ECV was successful in 96% of patients who were on ACE inhibitors before start of the present episode of AF compared to 80% of the patients not pre-treated (p = 0.04). After 1 month of follow-up 49% of the pre-treated patients and 50% of those not pre-treated with ACE inhibition were still in sinus rhythm (p=ns). Multivariate analysis showed that pre-treatment with ACE inhibitors and a smaller left atrial size were independent predictors of successful ECV (OR = 5.8, C.I. 1.3–26.1, and OR = 5.6, C.I. 1.2–25.3, respectively).

**Conclusions:**

Pre-treatment with ACE inhibitors may improve acute success of ECV but does not prevend AF recurrences.

## Background

Persistent atrial fibrillation (AF) is difficult to treat. In the absence of class I or III antiarrhythmic drugs sinus rhythm is maintained in only 30–50% of patients during the first year after Direct Current electrical cardioversion (ECV)[1,2]. Furthermore, even following an aggressive approach with repeated ECVs and use of prophylactic drugs, arrhythmia-free outcome is still poor: only 39% of patients maintain sinus rhythm during two years of follow-up[1,2]. Notwithstanding the recent results of AFFIRM and RACE showing no beneficial effect of rhythm control over rate control a rhythm control strategy may be indicated in severely symptomatic patients and those with a tachycardiomyopathy[3]. In recent years research has focused on the atrial remodeling processes that are induced by AF itself and that trigger the arrhythmia to become sustained: "AF begets AF"[4]. One of the remodeling processes induced by AF is fibrosis. Fibrosis causes dispersion of conduction, which, in its turn is related to inducibility of AF[5]. The renin-angiotensin system seems to play an important role in the development of fibrosis in heart failure. It was shown that pre-treatment with enalapril may attenuate atrial fibrosis and conduction abnormalities in a canine model of heart failure, and the occurrence of AF in patients with left ventricular dysfunction [5-7]. A recent experimental study showed that angiotensin II blockers may prevent electrical remodeling when started before start of AF[8].

In the present study we report on the effects of ACE inhibition on the outcome of ECV and the prevention of early recurrences after ECV of persistent AF.

## Methods

One hundred-seven consecutive patients with persistent AF, defined as the presence of AF for at least 24 hours were included in this study[9]. ECV was performed according to a previously described step up protocol[10]. Successful ECV was defined as the presence of sinus rhythm for at least 4 hours after ECV. No difference was made between unsuccessful ECV due to shock failure or due to an immediate recurrence of AF (within 2 minutes after successful ECV). ACE pre-treatment was defined as use of ACE inhibitors before *onset *of the current AF episode. Most patients on ACE inhibitors used these drugs for hypertension or congestive heart failure. To make sure that all patients were not completely remodeled at the very moment of start of the current episode of AF (and thereby verifying the fact that they were treated with ACE inhibitors before the process of electrical remodeling started), only patients with at least 1 month sinus rhythm before the current episode of AF were included in this study. Duration of AF was determined as precisely as possible by previous electrocardiograms, 24-hours Holter registrations, and by the patient's history. None of the patients were on class I or III antiarrhythmic drugs neither at the moment of ECV nor during follow-up.

### Statistical analysis

Quantitative variables were compared between groups using a two-tailed t-test for normally distributed variables or a Wilcoxon two-sample test for skewed distributed variables. For qualitative variables (categorical or ordered), group differences were evaluated using a Fisher's exact test or a Chi-square test. Accordingly, baseline characteristics are given in mean ± SD, median and range (min-max) or percentages.

To determine the predictive factors for successful ECV, an univariate logistic regression analysis was performed using the relevant baseline predictors. Variables with a p-value < 0.20 were selected for the multiple logistic regression analysis to derive a model with statistically significant predictors, by using a backward selection method. All p-values are two-sided and a p-value of < 0.05 was considered statistically significant. SAS version 6.12 (Cary, NC) was used for all statistical evaluations.

## Results

Baseline characteristics are listed in table 1. Twenty-eight patients (26%) were treated with ACE inhibitors *before *start of the current episode of AF (pre-treated patients). In the latter group, ECV was successful in 96% while in the group of patients not pre-treated with ACE inhibitors before start of the current AF episode only 80% had a successful ECV (p = 0.04).

**Table 1:** Baseline characteristics

	All	Successful ECV	Unsuccessful ECV	P
	N = 107	N = 90	N = 17	

Duration in days, median (range)				
current episode	115(71–175)	113 (61–175)	126 (81–189)	ns
total AF duration	144 (95–232)	142 (86–213)	160 (102–340)	Ns
Number of previous ECVs, N(range)	1 (0–5)	1 (0–5)	1(0–3)	Ns
Underlying heart disease * (%)				
Left ventricular dysfunction	21%	25%	0%	0.02
Hypertension	30%	32%	17%	ns
Valve disease	19%	19%	22%	ns
Coronary artery disease	22%	21%	28%	ns
Lone AF	16%	16%	15%	ns
Echocardiography (mm ± SD)				
LA parasternal long axis	47 ± 8	47 ± 9	47 ± 8	ns
LA 4 chamber view long axis	67 ± 9	67 ± 10	67 ± 6	ns
LA 4 chamber view short axis	46 ± 8	46 ± 9	43 ± 5	ns
RA 4 chamber view long axis	62 ± 8	62 ± 8	63 ± 7	ns
LVEDD	50 ± 8	50 ± 8	50 ± 7	ns
LVESD	35 ± 10	35 ± 10	33 ± 9	ns
Medication				
Beta blocker	49%	50%	48%	ns
Calcium channel blocker	36%	36%	37%	ns
Digoxin	47%	46%	49%	ns
ACE pretreatment	26%	30%	6%	0.04
Diuretics	31%	31%	29%	ns
Angiotensin receptor blocker	1%	1%	0%	ns

* more then 1 entity per patient

AF = atrial fibrillation, ECV = electrical cardioversion, LA = left atrium, RA = right atrium, LVEDD = left ventricular end diastolic diameter, LVESD = left ventricular end systolic diameter

In general, patients treated with ACE inhibitors before start of the current episode of AF had more evidence for heart disease than those who were not pre-treated. Prevalence of hypertension (46% versus 24%, respectively, p = 0.03), and left ventricular dysfunction (40% versus 14%, respectively, p = 0.01) was significantly higher in the pre-treated group in comparison to those who were not pre-treated with ACE inhibitors. (Table 2) Furthermore, there was a trend towards a higher prevalence of coronary artery disease in the pre-treated group compared to the not pre-treated group (36% versus 18%, respectively p = 0.07).

**Table 2:** Baseline characteristics divided in ACE pre-treatment and no ACE pre-treatment.

	No ACE pre-treatment	ACE-pre-treatment	P
	N = 79	N = 28	

Duration in days, median (range)			
current episode	121 (74–178)	96 (59–135)	ns
total AF duration	161 (105–247)	117 (63–160)	ns
Number of previous ECVs, N(range)	1 (0–5)	1(0–3)	ns
Underlying heart disease * (%)			
Left ventricular dysfunction	14%	40%	0.01
Hypertension	24%	46%	0.03
Valve disease	19%	21%	ns
Coronary artery disease	18%	36%	0.07
Lone AF	17%	14%	ns
Echocardiography (mm ± SD)			
LA parasternal long axis	46 ± 7	50 ± 10	0.03
LA 4 chamber view long axis	66 ± 9	67 ± 12	ns
LA 4 chamber view short axis	45 ± 8	49 ± 10	ns
RA 4 chamber view long axis	61 ± 8	64 ± 7	ns
LVEDD	49 ± 7	54 ± 8	0.01
LVESD	34 ± 9	38 ± 12	ns
Medication			
Beta blocker	51%	47%	ns
Calcium channel blocker	35%	39%	ns
Digoxin	45%	51%	ns
Diuretics	29%	34%	ns
Angiotensin receptor blocker	1%	0%	ns

* more then 1 entity per patient

AF = atrial fibrillation, ECV = electrical cardioversion, LA = left atrium, RA = right atrium, LVEDD = left ventricular end diastolic diameter, LVESD = left ventricular end systolic diameter

An additional 21 patients received ACE inhibitors *after *start of the current episode of AF because of either insufficiently treated hypertension or left ventricular dysfunction newly documented with echocardiography. Success of ECV in these patients was comparable to patients who were not treated with ACE inhibitors at all (80% and 82%, respectively). Use of beta adrenergic receptor blockers, calcium channel blockers or digoxin, started before the present episode of AF or not, did not influence success of ECV.

**Figure 1:**
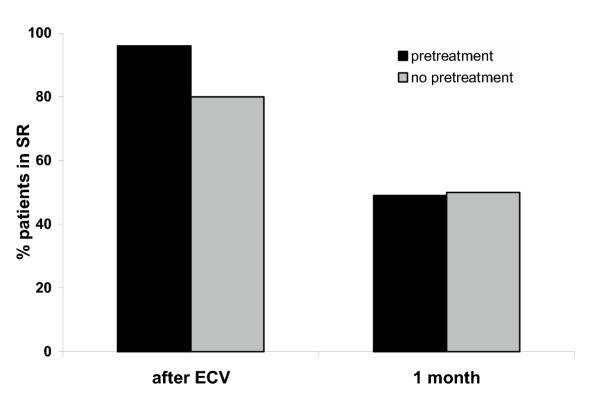
Effect of pretreatment with ACE-inhibitors before start of AF.

After 1 month of follow-up 49% of the pre-treated patients compared to 50% of those who were not pre-treated with ACE inhibition were still in sinus rhythm (Table 3, Figure 1).

**Table 3:** 

	All	No ACE-pretreatment	ACE-pretreatment	P
Successful ECV (%)	84%	80%	96%	0.04
SR at 1 month follow-up (%)	50%	50%	49%	ns

ECV = electrical cardioversion, SR = sinus rhythm

Multivariate analysis showed that pre-treatment with ACE inhibitors and a smaller left atrial size were independent predictors of successful ECV (OR = 5.8, C.I. 1.3–26.1 and OR = 5.6, C.I. 1.2–25.3, respectively).

## Discussion

### Main results

This post-hoc retrospective analysis shows that use of ACE inhibitors before the onset of AF enhances acute ECV outcome but it does not improve maintenance of sinus rhythm. Furthermore, when ACE inhibitiors are instituted later, i.e. after the start of AF, it does no longer improve success of ECV.

### Effect of ACE-inhibition on structural remodeling

ACE inhibitors reduce the incidence of AF in patients with left ventricular dysfunction[7,6]. This may be related to the protective effects of ACE inhibitors, which help to maintain atrial integrity and attenuate fibrosis[5]. In the present study pre-treatment with an oweveHowACE inhibitor (i.e. use of ACE inhibitors before onset of the arrhythmia) improved acute outcome of ECV. In view of the above this suggests that ACE inhibitors may prevent or diminish AF induced structural remodeling. These clinical findings are compatible with experimental findings showing that ACE-inhibition could attenuate heart failure induced atrial functional remodeling and fibrosis in dogs[11]. In atrial tissue from AF patients an ACE-dependent increase of activated extracellular signal-regulated kinase (Erk) type 1 and 2 was found, which may contribute to the development of atrial fibrosis during AF[12].

### Effect of ACE-inhibition on electrical remodeling

In 1995 it was shown that AF induces several electrophysiological changes, called electrical remodeling[4]. Experimental data show that ACE inhibition prevents short-term (< 2 hours) tachycardia-induced atrial electrical remodeling[8,13]. However, enalapril could not attenuate or prevent the long-term (7 days) effects of tachycardia on remodeling[8]. Several studies have investigated the role of calcium channel blockers on electrical remodeling. In these studies it was also shown that although there was a short-term prevention of electrical remodeling, calcium channel blockers did not have a long-term protective effect on electrical remodeling [14-16].

### Effect on AF recurrences

Madrid et al. investigated the role of the angiotensin II type 1 receptor antagonist irbesartan in amiodarone treated patients[17]. Persistent AF patients were randomized to either treatment with amiodarone alone or treatment with amiodarone in combination with irbesartan. Drug treatment was started at least 3 weeks before cardioversion but *after *the start of AF. No differences were found in electrical cardioversion outcome between the two treatment groups which is in contrast to the present study. This may relate to the fact that all patients were in AF at the very moment of start of irbesartan. Two months after cardioversion it appeared that patients treated with irbesartan and amiodarone had a significantly lower AF recurrence rate compared to amiodarone alone treated patients, 15% versus 37%, respectively (p = 0.007). This difference was maintained during a median follow-up of 254 days. According to their figure 2, the benefit of irbesartan (in combination with amiodarone) was mainly achieved by reduction of recurrences after the first two weeks after cardioversion. An earlier study on the role of ACE inhibitors in patients with heart failure and AF showed a trend towards more patients maintaining sinus rhythm after ECV when instituted on lisinopril in comparison to patients not treated with lisinopril[18]. In the present study no difference in recurrence rate could be found between patients with and without ACE-pretreatment.

## Limitations of the study

This was a non-randomized and post-hoc analysis. This implies that all findings can only be used to generate hypotheses. However, this is the first clinical study showing that ACE-inhibition initiated before start of AF enhances direct ECV outcome.

Only a very small amount of patients was on angiotensin receptor blockers. Whether the effect of pretreatment with angiotensin receptor blockers on cardioversion outcome would be the same as the effect of pretreatment with ACE inhibitors could not be investigated in this study. However, in this context the results of the study of Madrid et al. are encouraging [17].

## Conclusions

Pretreatment with ACE-inhibitors significantly improves acute outcome of ECV when initiated before the onset of AF but it does not lead to better maintenance of sinus rhythm. When ACE inhibitiors are, however, instituted later, i.e. after the start of AF, it does no longer improve success of ECV.

## Competing interests

The author(s) declare that they have no competing interests.

## Authors' contributions

TVN carried out the study and drafted the manuscript, HC designed the study, and interpreted the data, MB participated in the draft of the manuscript and interpreted the data, DVV participated in the draft of the manuscript and interpreted the data, IVG participated in the design of the study and interpreted the data. All authors read and approved the final manuscript.

## Pre-publication history

The pre-publication history for this paper can be accessed here:


